# “I Was Given Three Marks and Told to Buy a Porsche”—Supervisors’ Experiences of Leading Psychosocial Safety Climate and Team Psychological Safety in a Remote Academic Setting

**DOI:** 10.3390/ijerph191912016

**Published:** 2022-09-22

**Authors:** Kirsi Sjöblom, Jaana-Piia Mäkiniemi, Anne Mäkikangas

**Affiliations:** 1 Work Research Centre, Tampere University, 33014 Tampere, Finland; 2 Finnish Institute of Occupational Health, 00032 Helsinki, Finland

**Keywords:** psychosocial safety climate, PSC, team psychological safety, leadership, remote work, academia, university organizations, occupational health, well-being, COVID-19

## Abstract

This study examines leading psychosocial safety climate (PSC) within the organization and psychological safety in teams in remote work conditions caused by the COVID-19 pandemic. These topical working life phenomena have an essential role in health, well-being and productivity in today’s working life, but they have rarely been studied in remote work context. A total of 26 supervisors and leaders at three Finnish universities participated in semi-structured interviews. The data were analyzed using qualitative content analysis, resulting in four main categories: supportive and challenging aspects of leading psychological safety and well-being, supportive and challenging aspects of organizational psychosocial safety climate leadership, support for working as a supervisor, and characteristics specific to working in academia. The results indicate that leading psychological safety remotely requires more time, deliberation and intentionality than when working face to face, and that the role of remote interaction is underlined in it. As to PSC, it is important to improve the cohesion in leading psychological safety and health in academic organizations. How PSC is led in the organizations affects not only the general psychosocial working conditions, but also the possibilities for good leadership of psychological safety in smaller units in the organization. The study makes a novel contribution especially in understanding (1) leadership of PSC and psychological safety in remote work conditions, and (2) the reciprocal relations between leading psychological safety and well-being at the organizational level and the team level.

## 1. Introduction

Developing good practices for sustainable work and supporting employee well-being are increasingly important in today’s working life [1,2]. Leadership practices have a pivotal role in this [3]. Although remote work (i.e., work carried out outside the main office) [4], is not a novel phenomenon as such; during the extended enforced remote work period necessitated by the COVID-19 pandemic, the number of remote workers increased significantly [5]. While some leaders and employees had remote work experience preceding the pandemic, for many, the remote work circumstances and practices came as a sudden and unexpected challenge. In addition, before the COVID-19-pandemic, it was more common to work multi-locationally [6,7], that is, combining remote working and working at the office on voluntary basis. During the pandemic many had to work entirely remotely [7,8], which has brought new challenges compared to combining remote work with working face to face. Remote work conditions are likely to continue to be a central way of working even after the pandemic subsides [9] and therefore there is an intensified need to understand and develop workplace practices specific to remote work [7,9,10].

Since leadership practices are typically studied from the perspective of subordinates, in the present study we concentrate on leaders’ perceptions and experiences. We focus specifically on the leaders’ perceptions of good practices in leading psychosocial safety climate (PSC; i.e., organizational climate for employee psychological safety and health) [11], team psychological safety (i.e., team’s shared belief that the team is safe for interpersonal risk-taking) [12], and occupational well-being, as these aspects have an essential role in not only health and well-being but also in productivity at work [13,14,15]. We examine leaders’ views on leading psychological safety, both on their own part on team- or unit-level, and in their perceptions, experiences, and ideas on organizational level leadership practices related to psychosocial safety climate.

Every organizational context has its own characteristics which are also reflected in leadership practices. We focus here on the Finnish higher education system. In this context, supervisory duties often come at short notice along with the granting of external project funding and hiring employees for this; the supervisors typically have many other core responsibilities and roles in their jobs and the supervisory role is something additional, thus, the time allocated for supervisory work or the training related to it may be very limited [16,17,18]. In addition, the Finnish university system has encountered many changes [19], and currently job contracts are often fixed term for employees and even for leaders themselves, which makes it challenging to develop long-term collaboration and relationships with subordinates and teams. Overall, the work in academia tends to be fairly intensive and competitive, and the standards set for it are high [20,21,22]. In comparison to many other nationalities, Finnish academics report high levels of both stress and satisfaction [23] and the level of work engagement is high [19]. These aspects, along with the generally highly demanding nature of academic work, offer a relevant context for studying leadership related to psychosocial safety and well-being.

This study contributes to the existing literature on remote leadership in the following *three* ways: *first*, in spite of a vast body of research on psychological safety and leadership in general, little is so far known about leadership practices that support psychological safety and well-being specifically in *remote working conditions* and empirical studies in particular are very rare. Furthermore, according to the existing literature, it is, in fact, a challenge to maintain a sufficient level of psychological safety and well-being in entirely remote working conditions; this requires intentionality and strategy [24,25,26]. Therefore, it is crucial to gain more understanding on the topic.

*Second*, leadership is most often studied from the perspective of subordinates [3,27,28,29,30], and during the pandemic, leaders’ experiences of the changes and challenges occasioned by the sudden shift to working from home have likewise been studied much less than those of employees [31]. There is reason to believe that, based on their own experience of leadership during the pandemic, leaders are able to point out elements that are essential in leading psychological safety in remote work. Furthermore, it has been argued that research on psychological safety and leadership would benefit from examining the topic from multiple perspectives [14,32]. Therefore, this study examines the topic from *the perspective of the leaders* themselves.

*Third*, *qualitative studies* examining psychological safety are generally, rare, probably due largely to the fact that psychological safety is most often studied quantitatively by using questionnaires measuring team psychological safety [12] and psychosocial safety climate [11,33]. Qualitative methods offer a powerful set of tools to explore novel research topics and phenomena, or established phenomena and theories in new contexts, related to which the participants are likely also to raise unforeseen aspects and angles [34,35,36]. Furthermore, qualitative methods are ideal to study current organizational changes and newly emerging topics such as new ways of working [37,38], of which the remote leadership and psychological safety studied here are topical examples. *Fourth*, there are so far very few empirical studies examining *team psychological safety and psychosocial safety climate concurrently* [39,40]. This study makes an important novel contribution in bringing them together and shedding light on the interconnections between these two focal working life phenomena.

### Leading Psychological Safety during Enforced Remote Work

Although prioritizing employee well-being and psychological health has been essential in working life since long before the COVID-19-pandemic, the exceptional circumstances it brought about have challenged employees’ well-being in new ways and further underlined the importance of making employees’ health and well-being a top priority within organizations. Enforced remote work has challenged accustomed ways of working and collaboration in numerous ways, and leadership is no exception [41,42]. In remote work conditions, everyday contact between leaders and employees is typically much less frequent than when working within the same physical premises, which has certain consequences for both the practicalities of work as well as for communication, interaction, and well-being [43,44]. Earlier studies have also found that employee self-leadership skills are particularly important in a remote work setting [45,46,47], which also changes the dynamics of leadership: employee autonomy is even more accentuated, and each employee needs to play a much bigger role in guiding their own work on a daily basis than when working in more traditional workplace facilities.

It has been noted that building psychological safety in virtual teams entails certain specific challenges and requires effort and strategy, and that this can be facilitated by utilizing specific remote communication strategies, functions, and tools [24,25,26]. At the organizational level, a recent study showed that the extent to which the remote work necessitated by the corona crisis affected employees depended heavily on how organizations responded to the situation [48]. Another study found mixed results on PSC both increasing and decreasing over time during the COVID-19 pandemic and concluded that the context of the pandemic has highlighted the importance of a positive workplace climate and working conditions [49]. Indeed, remote work appears to even further underline the necessity of fostering trusting relationships and psychological safety [24], making it vital for research to acquire more understanding on psychological safety in remote work context.

In this study, we are particularly interested in leaders’ perceptions and experiences of leading psychological safety in remote work conditions. According to earlier research [12,13,50], psychological and psychosocial safety can be studied and led at both *team level* and *organizational level*, and in this study, we examine leaders’ perceptions of both. *Team psychological safety* is a group-level phenomenon defined as a shared belief held by members of a team that the team is safe for interpersonal risk-taking [12]. It includes several team-climate related aspects such as believing that team members will not reject each other for being themselves, and experiencing that team members care about each other as individuals, have positive intentions to one another, and respect the competence of others [12,15]. Team psychological safety has proved to have a pivotal role in many positive workplace behaviors, aspects of well-being and job attitudes, such as learning behavior, work engagement, and job satisfaction [14,15].

Numerous studies have examined the role of leadership in team psychological safety and found that supportive leadership behaviors affect various work outcomes through psychological safety at both the individual and team level [15]. Supportive leadership behaviors, such as leader inclusiveness [51,52], support [53], and trustworthiness [54] have been shown to decisively influence individual employees’ perceptions of psychological safety, which in turn affects positive employee outcomes such as work engagement, job performance, creative work, and voice behaviors [15]. On the other hand, it has been pointed out that the boundary conditions of psychological safety remain an understudied area, particularly so in the case of the role of leadership: leadership matters in fostering psychological safety, but in what conditions does it matter most [14,32]? In this study, we focus on the context of academic remote work and the exceptional period of crisis.

Besides the team and immediate supervisor having a focal role in experienced psychological safety, upper management also has a vital role in setting the tone for the importance of well-being-related issues within the organization and in enabling the necessary practices for supporting well-being. *Psychosocial safety climate* (PSC) refers to an organizational climate for employee psychological safety and health [11]. It is defined as the “politics, practices, and procedures for the protection of psychological health and safety of workers” [13]. Psychological safety climate comprises four content domains: (1) senior management’s support for and dedication to stress prevention through involvement and commitment; (2) management’s assigning priority to psychological health and safety versus productivity goals; (3) organizational communication, that is, the organization listens to contributions from employees; and (4) organizational participation and involvement, for example, participation and consultation occurs with unions and with representatives of occupational health and safety [11] (p. 355). As a result, an impression is conveyed to the employees as to what kind of role and importance psychological health and safety has in organizational leadership.

PSC can be seen as the origins of psychosocial working conditions—it creates the framework for well-being within the organization and affects the psychological health and engagement of employees [13]. In prioritizing employee well-being, PSC has the potential to both increase job resources (e.g., supportive organizational climate) and decrease job demands (e.g., stress and burnout). In this way, PSC is linked to the job demands-resources (JD-R) framework [55], which states that high job resources are associated with positive employee outcomes such as work engagement, and high job demands with negative outcomes such as stress and job burnout. On a practical level, examples of PSC-supportive leadership actions can be arranging regular opportunities for employees to have their well-being related experiences and views heard and taking the necessary actions of intervention and prevention, such as adjusting resources or providing well-being related support.

In this study, we were interested in academic leaders’ views on leading psychological safety remotely: on the one hand, their own practical experiences as remote leaders, in the exceptional conditions of enforced remote work caused by the COVID-19 pandemic. On the other hand, we were interested in how in their opinion psychosocial safety climate had been led during that time and what could be done better with regard to leading psychological safety and occupational well-being within the organization in the future. Our research questions were:

Research question 1. What aspects do supervisors and leaders view as central in leading team psychological safety and well-being in remote working conditions?

Research question 2. What aspects do supervisors and leaders view as central in leading psychosocial safety climate and well-being at the organizational level in remote working conditions?

## 2. Method

### 2.1. Participants

The data used in this study were collected as a part of the research project “Safely remotely—occupational well-being and its management in telework”, funded by the Finnish Work Environment Fund. The overall focus of the research project was leadership practices and well-being during enforced remote work caused by the COVID-19 pandemic. The participants (*n* = 26) of the current study first responded to a web survey that concerned occupational well-being during enforced remote work. It was sent to the work email addresses of 12,120 employees of three Finnish universities through mailing lists. The survey was available from April 12 through 3 May 2021, and altogether 3543 employees participated (response rate 29%) [47]. Respondents working in a supervisory capacity or leading position were asked if they were willing to take part in an interview focusing on leadership during the enforced remote work period. Altogether 168 participants indicated their willingness to participate in an interview. Out of this group, 75 participants were randomly selected, and out of this remaining sample, the final participants were selected manually in order to ensure the diversity and representativeness of the sample in terms of university, position, experience, field, and gender.

Of the interview participants (*n* = 26), 46% were immediate supervisors (e.g., research group or team leaders), 39% were leaders or heads of the unit/center or similar, and 15% were director/dean level. The average age of the participants was 48.6 years (SD = 6.0) and 62% were women. Average work experience in the current position was 6.5 years (SD = 6.3). The participants represented different faculties and units at the universities: engineering; information technology and communication sciences; medicine, health technology, and health sciences; natural sciences; social sciences; and university support services.

### 2.2. Data Collection

All interviews were conducted by the first author, who is a researcher and a psychologist. The interviews were designed together by all authors and discussed during the process in order to gain multiple perspectives on conducting them. In addition, prior to the interviews, two test interviews were conducted to confirm the suitability of the questions and the continuum of the interview.

The main topics of the semi-structured interviews were (1) orientation and context of the interview (job description, supervisory tasks, and role as a supervisor/leader), (2) leader/supervisor well-being during the pandemic (personal experiences during the enforced remote work period), (3) leading psychological safety and well-being remotely (general experiences, challenges, best practices, needs for support), (4) psychosocial safety climate and well-being within the organization (personal experiences and viewpoints), and (5) assessment and future (experiences of success and potential for improvements regarding leading psychological safety and well-being, suggestions for improvement of organizational practices, other issues raised by the interviewees). The structure and questions of the semi-structured interviews are presented in more detail in Appendix A. The interviews were conducted online via Teams application as video interviews and lasted 45–60 min. They were carried out in October-November 2021. The interviews were recorded and transcribed verbatim for the purposes of the analysis.

### 2.3. Ethical Considerations

The study was conducted according to the guidelines of the Declaration of Helsinki, the Finnish National Board on Research Integrity, the EU General Data Protection Regulation (EU 2016/679) and the Finnish Data Protection Act (1050/2018), which stipulates the requirements for ethically sustainable data collection and storage. The study was approved by the Directors of Human Resources of the universities studied. Informed consent was obtained from all participants. Prior to the interview, the participants received by email a written communication on confidentiality, anonymity, and the voluntary nature of participation, as well as the background for the study. At the beginning of the interview these issues were explained once more.

### 2.4. Data Analysis

The data were analyzed following the main phases of qualitative content analysis [56,57,58], which is widely used in research on health and well-being. Its main advantages include the ability to condense extensive data into more concise and clear information in a systematic way.

ATLAS.ti 22 software was used for coding the interviews. The first author, who also conducted the interviews, first read through the transcripts and anonymized them all. In the preparation phase, the material was read through multiple times and notes were made along the way. Next, in the coding phase, codes describing the content of the expressions were formed based on the research questions and the theory on psychological safety and psychosocial safety climate (topic-oriented coding) [59], but also data-driven, remaining open to topics emerging from the interviewees. In the following categorization phase, the codes were compared, combined, redefined, and finally classified according to similarities in meaning into subcategories, generic categories, and main categories. Additionally, the contents and relations in these categories were further analyzed and redefined, and as a result, the final four final main categories were formed.

## 3. Results

The qualitative content analysis resulted in four main categories: (1) supportive and challenging aspects of leading psychological safety and well-being, (2) supportive and challenging aspects of organizational psychosocial safety climate leadership, (3) support for working as a supervisor, and (4) characteristics specific to working in academia (see Table 1). The two latter categories arose as central and essentially related to leading psychological safety and well-being in the university context. The main categories, generic categories, subcategories, and their interconnections are described in the following and illustrated with excerpts from the interviews.

### 3.1. Supportive and Challenging Aspects of Leading Psychological Safety and Well-Being

#### 3.1.1. Supportive Leadership Practices for Enhancing Psychological Safety in the Remote Working Context

According to the analysis, immediate supervisors aimed to support the employees’ psychological safety and well-being in remote working context by emphasizing trust, investing in remote interaction, being more deliberate about different forms of interaction, cultivating authenticity, nurturing an environment that accepts and even encourages mistakes, and by ensuring different aspects of the psychological and physical well-being of both the employees and themselves. Some of these practices are general in supporting psychological safety in any context whereas some are specific to the remote work conditions.

In order to support psychological safety in remote work circumstances, many participants emphasized practices aiming at good quality of interaction and fostering trust. Examples of these included expressing interest in how the employees were doing, making time to regularly contact employees individually either by phone or video meeting, making oneself available and encouraging employees to be in touch, generally communicating rather more than less, and connecting with as rich media as possible to maintain the connection and to avoid misunderstandings (e.g., choosing video or phone call over email). Many participants emphasized the importance of also connecting informally with the employees and within the team, not just around work and tasks, and some utilized novel, remote work-specific means to do this, such as creating different kinds of interaction channels in Slack, Teams or Whatsapp applications. Overall, the role of remote interaction was underlined in supporting psychological safety, and many participants pointed out that maintaining a good connection with employees in remote work conditions requires much more effort and time than when working face to face.

*On Monday mornings, we’ve had a meeting half past eight, sort of such that the week gets started off well, and very informal so we’re definitely not presenting any results or such, more like if someone has something on their mind or something good to tell others, - - (so) that people meet with each other and remember who [laughing], who we’re working with.*

*It has indeed required more initiative from the supervisor, which is perhaps difficult for me, since I’m really quite an introverted type. But simply so that I have put it into my calendar and I’ve called the employees, asking how everything is and how they are doing. - - some are such that they don’t get in touch themselves and they are there in the meetings but if they come, at least they have their cameras off and they don’t say anything. In a way it’s really easy to become marginalized and to fall behind. Because of that these kinds of personal calls have been important, so that you see how people are.*

*In my opinion, you need to think much more closely about what you are doing and what the aim of those encounters is.*

While many participants stated that building trust and creating or maintaining a good connection was more challenging remotely than face to face, as an exception, one interviewee mentioned that building trust within the team had succeeded better during enforced remote work than previously since their team was normally physically dispersed on different campuses, so the remote conditions had actually brought them together into one shared space. Others mentioned that the new ways of remote communication had enhanced the active role and voice of all participants better than the traditional face-to-face meetings.

Many participants pointed out that, especially in remote work conditions, trust not only needs to be cultivated, but it is also a necessary starting point for the work and the supervisory relationship in the first place. Without it, collaborating remotely is simply not possible, and it is also a core element in supporting the experience of psychological safety:

*Trust in-, that everyone sort of knows their own goal, but the journey there is for everyone to define-, or they get to affect how they do things or reach goals, and so somehow it has brought us that mental safety perhaps the most, that everyone is important, everyone is a valued employee and everyone’s view, it is like everyone is the best expert regarding their own job, that has sort of brought it to us the most.*

Some participants mentioned bringing their own personality into work and being themselves as leaders so as to encourage others to be able to do the same. Some described encouraging employees to accept and even comprehend the value of making mistakes, and pointing out that they themselves are not infallible also. They emphasized that they also did not know all the answers and saw working with employees rather as a process of shared learning and collaboration. On the other hand, some participants mentioned supporting employee well-being and safety by supporting them in their tasks and the substance of work, and by being available to help in case of problems while others mentioned remembering to celebrate successes together.

*As to that psychological safety, apparently, I am very permissive [laughing], like in my opinion people are allowed to and even should make mistakes because they are perhaps also one way to learn. As a matter of fact, we have a fair number of those Erasmus students, they are in their third or fourth year, and especially those coming from southern European countries have been thanking me for being allowed to make mistakes over here [with a laugh]. - - I always say that we are learning together here, I do not have those ready-made answers.*

*Such that when we’ve got to finish some job, for example an article published, it has been brought up and highlighted, like hey this hero has got this kind of a thing done now, and then in our Slack forum everyone congratulates and so on. - - (gathering and having a glass of sparkling wine) has been dropped or replaced by these kinds of celebrations on Slack or something like that.*

Some participants pointed out that the precondition for them to be able to be supportive and accepting of everything that the employees expressed and shared was to have similar support for oneself as well, and likewise, in order to support employee well-being it is essential to take care of one’s own well-being first.

*When I’m able to reset the situation when I get vexed, it helps me, - - I hope that, all of our employees need to have that kind of steam valve, that they can let out whatever is bugging them about me. They can say it to me directly too and some have, too, and it’s terribly fruitful. And I can’t say that I wouldn’t be offended sometimes, but I can take it just fine.*

*I suppose there are many things like, starting from the angles of one’s own well-being and coping, like how you separate work and free time yourself, and how you’re able to disengage, because currently I notice it clearly that I am genuinely very, very tired, and it affects resilience and many sorts of things then in your life. It is perhaps again one of those basic lessons, take care of yourself first before you take care of others.*

Many participants took the view that the enforced remote work came with many potential threats to well-being and described giving their employees explicit well-being-supportive guidelines, such as encouraging them to take enough breaks during the day or meet each other, and ensuring that the employees had the needed equipment and support at their home offices.

*Communicating that too, that we have the right, and in fact a duty too, to reserve those breaks there within the working day. Especially sitting here at home, it is so much easier to immerse oneself in front of the computer, when you don’t have that person next to you saying hey, should we go and have lunch. And then I’ve tried to be a bit of an example myself in that too, having booked those lunch breaks.*

*I think that it has had an effect too that I say out loud that hey, I don’t idealize you working in the evenings and I don’t expect it, and I don’t think that it’s reasonable to try to do too much.*

Interestingly, although many interviewees mentioned numerous psychological safety supportive actions and some were very skilled in this, there was clearly considerable variation in how familiar the supervisors and leaders were with the phenomenon and how easy it was for them to discuss their views on the matter. More general aspects of occupational well-being, on the other hand, appeared already well established across all participants.

#### 3.1.2. Challenges in Leading Psychological Safety and Well-Being Remotely

The most prevalent comment on leading psychological safety and well-being in remote work circumstances was that it was very challenging. According to the data, it was challenged by limited interaction and the consequent uncertainty of the employees’ situations and well-being, workload, and lack of time for maintaining sufficient contact with the employees, which required more time remotely. In addition, increased intensity of work, and varying employee competence in managing their own work and well-being in the highly autonomous remote working conditions were experienced as challenges to leading psychological safety and well-being remotely, as we describe in more detail in the following.

Many participants pointed out that it was very hard or even impossible to know how the employees were really doing at the other end of the line, and many descriptions of how the remote working period had gone in this regard were tinged with uncertainty. Many leaders worried a lot about their employees and found it stressful that they did not know if they had done enough. They noted that at times their impressions of employees’ well-being could be rather deceptive:

*When I see those people here, I can already tell based on their body language if they are doing well or not. - - You can tell based on the expression, but then, with the email only, I send a message asking how they’re doing. Then everyone’s going to be like yeah, everything’s going alright. - - But for example, if one of my subordinates had a drinking problem, it’s usually the kind that you don’t easily bring up. Usually it comes out a little too late since people try to hide it. And if we think, having worked about a year remotely, for example. Frankly, they could be even shooting heroine there if -, if you have for example that one hour-long meeting per week, of course you can prep yourself for it. - - So you’ll be able to be quite sharp and it works just fine. Compared to meeting live every day, it doesn’t show. So in my opinion it’s completely unrealistic that you could, especially this idea of early intervening, which is discussed a lot at the university. In my opinion, remotely, if not impossible, it is at least very hard.*

*One gets a rather erroneous impression that everything seems to be rolling along awfully well. We have all the equipment we need, and the capabilities required for them, it works well. On the average very well indeed, but then when you finally get to the top of it, the experience of loneliness for some has actually been quite rough.*

*But I have to say that for me it came as a surprise in one meeting that they started crying and such. And I had no idea that we were in such a bad situation. So that is maybe a good example of how you may answer by email and say yeah, doing okay, no problem. Doing work here, going well.*

*I kind of still constantly struggle with it a bit, if I have been in contact with people enough, although we have been in touch and so, but… and I have contacted people on a low threshold and organized meetings and called and such but somehow it always makes you wonder, was it now -, how has this been with them, did they feel left alone at some point or how has it been and for that I don’t really have an answer.*

In addition to the limited interaction, many participants expressed concern about their employees because they realized that the enforced remote work period had posed specific challenges to well-being, for example the intensity of working days and the emphasis on productivity had generally increased and the connection within teams decreased:

*- - now during the pandemic I need to schedule a Zoom meeting six days away where I can see this person, and even then I somehow don’t get an honest picture, because the message is mediated like this, the kind of understanding that happens within a work community, it disappeared. And of course we set up all kinds of Zoom coffee meetings and such, but they’re not the same. So to some extent it feels like people have been working more efficiently, because they’ve been able to concentrate, but it has happened at the expense of the work community and at the expense of their own well-being.*

*We are also all, or this whole gang, quite performance-oriented, we take care of it and we’re efficient and then no one has the energy for the chitchat, it just doesn’t happen remotely like this which on the one hand is good but bad on the other. - - This is more like we have this agenda, let’s get this done.*

*The certain kind of informal being together, it’s missing. Somehow, I don’t know how to create it. Maybe it would require more of those one-on-one encounters where somehow one should dare to put oneself out there more too. But in a certain way that trust, and the sense of relatedness among that group is built on, the kind of, not taking care of work-related things, but all the small things that would happen if we’d go for coffee together and chat about this and that together.*

For their own part, many participants mentioned the challenge of workload and time—leading psychological safety and well-being remotely required more time, but nothing had been removed from their normal tasks. Consequently, it was difficult for supervisors to have enough time to maintain the connection with the employees or in many cases it resulted in longer working hours and happened at the expense of their own well-being.

*Well, I would probably most need time. I mean, that I would also have time for stopping by at someone’s door when I come to work and ask how they’re doing and such. - - in a certain way it is creditable that we have training for this and that and for all kinds of things. But there is such an enormous amount of it, that if I really went to all of them, I wouldn’t have time for anything else anymore. - - in addition to just being trained to psychological well-being and such, in my opinion we should also have time to talk, because I find that it’s important. Time for the practical part specifically. That you treasure it with some kinds of actions, for example if it is possible sometime, to go for pizza together with everyone. All kinds of things, like let’s go exercise out of doors, let’s do something like this.*

*Even though I’m terribly busy, one can be in touch. - - then I quickly write it down on a post-it because I don’t remember anything since I have so much going on, and I’ve said that I have such a tough deadline on, I need to get this done, but why don’t I call you tomorrow. Then I’ve called the next day and we’ve had space to go through that issue calmly. I’ve made that space and ditched some other duties, said that I won’t come to this one if that’s been the only way to get that space, - - My own well-being is a little, on the risky side [with a laugh]. I have way too many working hours.*

Another aspect emerging in the interviewees’ experiences was the employees’ varying capabilities to manage their own work. It was difficult for the supervisor to support the well-being of employees lacking basic skills in managing their everyday work routines.

*But this remote work, whether you’re a supervisor or not, it requires a lot of self-leadership skills from all of us, on a whole different level than working at the workplace. And in my opinion for that maybe there is not enough wakening the people up to how each of us would self-improve. Because whether you’re a supervisor or a team member, it requires self-leadership skills just the same. And it requires, it is also on everyone’s responsibility to bring things up. So it also requires courage and skills too in how to drive this forward.*

### 3.2. Supportive and Challenging Aspects of Organizational Psychosocial Safety Climate Leadership

#### 3.2.1. Perceived Organizational Support for Psychosocial Safety Climate and Well-Being

Experiences of organizational support for psychosocial safety climate and well-being differed quite a lot across organizations. While some participants spoke highly of their organization’s practices related to this, challenging aspects were much more prevalent in the data. It appeared that in many cases organizational leadership practices regarding psychosocial safety climate were somewhat arbitrary and lacked cohesion within the organization.

Positive experiences included strong support and prioritization from the organization for well-being related aspects of work, for example establishing a new unit dedicated to advancing well-being at work, or having comprehensive means of support for both employees’ well-being and for working as a supervisor.

*Over here [information removed for purposes of anonymization] things are pretty much OK for us, I have a pretty straight line both to occupational health care, we have this chat that I can reach instantly on my cell phone, for example, and we also have an occupational well-being unit and from them I receive (help) immediately, I have been in touch with them.—so there are quite good ones (means of support). Now currently I am also taking part in leadership training, so I have peer support as well, so I have a pretty good situation and then we also have our own group here (within the team).*

*Yeah, and our current deans are pretty interested, which has helped a lot, for example they have, without any specific request, written blogs on well-being related topics - - It is quite something.*

Regular opportunities for interaction with top management and healthcare services regarding well-being related issues were also appreciated and found supportive:

*And then of course the rectors and the vice rectors visit different departments regularly. Occupational health care as well, so people are given opportunities for that (to be heard).*

*I can’t tell how it is in the eyes of a regular employee, but at least I as a supervisor experience that I get to go to these, I get to talk with the rector one on one every now and then, which is also a rather big change to what it used to be like sometime earlier.*

Explicit guidelines and support for well-being related practices from top management were viewed as important support for both individual well-being and for leading well-being in smaller units:

*That the director always said directly that hey, during daytime, go for a walk after lunch or take the dog out during the day, these kinds of concrete things, that this is okay and you’re allowed. It was really important, because then I could repeat it (to my team), that for real, each one of us, we can do this and let’s remember these breaks and the part of well-being, we get the same pay whether we sweat our guts out or not, and taking the breaks is more important than you might think.*

Some interviewees also pointed out that in their view the well-being-related policies had evolved a lot over the years and there had been many changes for the better in academia regarding how well-being-related issues are taken into account.

*Now there are good structures and it has been invested in, like I said, I have been a part of these things for something like 12 years, a lot of effort has been put into them and they are very easily available (referring to well-being-related support).*

#### 3.2.2. Organizational Practices Perceived as Hindrances to Psychosocial Safety Climate and Well-Being

In this data, experiences of organizational practices risking employees’ well-being were prevalent. These included inconsistency in well-being related policies, excessive workload, and lack of resources, straining reforms, little support for well-being-related supervisory work, and elements of uncertainty, as we describe in more detail below.

A common experience was that the organization’s well-being policies were inconsistent: the organization emphasized well-being support, e.g., by offering good occupational health services and training, but at the same time ignored issues and complaints about insufficient resources and unreasonable workload. Many noted that as a conclusion, these policies were mere window-dressing, not reality.

*A lot of talk and few actions. Unfortunately, it does seem a bit as if it remains at the level of ceremonial talk.*

*I would think that there is interest in these things and that people are listened to. So I don’t take a negative stand on it. But, maybe I do in the sense that the priorities of that area have not so far been very high.*

*In my view the university has the will and the way to take care of the employees’ psychological safety and well-being, but it’s a question of resources. If we have completely overburdened people in their jobs, it doesn’t help that they’re offered maybe slightly better pay or something else, you can’t buy more resources from an employee with money, instead there’s two ways: hire more people or reduce the duties. There is no other solution.*

*We were gravely under resourced, that [information removed for purposes of anonymization] sector, because there was an intense wrangle at the level of university management between different sectors on who gets what and how to share scarcity. As soon as it was possible, I discussed with the faculty management that we need to get a new person here, or else there is a risk of the other [information removed for purposes of anonymization] specialist leaving, or they get burnt out, two options. Either they leave or burn out.*

*I had it really challenging -, the situation within the team was, let’s say that the situation is still like I’ve been given three marks and told to buy a Porsche. And when I ask which parts of the Porsche I should leave at the garage since I can’t buy them all, I’ve been told oh no, those and the luxury version too, and I’m like, right.*

Similarly, many pointed out that the employees were burdened with many laborious reforms, also during the COVID-19 crisis. Some reported that the combination of lack of resources, excessive workload, and various constant reforms was beyond endurance and actually driving employees to burnout and illness, especially at a time of crisis whereas lightening the load would have been needed.

*But in the last organizational reform that was carried out, people got very little say. Our gang was put through the wringer. It was really rough, if I think about my own old team, there were people who were really unwell. They were downright ill. I did a lot of work to get them fit for work, and at first to a zero level so that you don’t need to be positive, but so that you wouldn’t be pissed off about it all the time, frankly. There were some really tough experiences. - - then someone said to us that you’d need some kind of debriefing, I said yes we really would, but there’s no time for that. As a supervisor I did what I could. - - Then we were sent to [laughing] to some kind of in-service training and it was pretty ridiculous. I tried to keep [laughing] a straight face and not to laugh or cry, when, really, we would have needed mental resources of a very different kind and that we would be listened to. It was downright dismal, that last reform.*

*I don’t really know if there’s anything that hasn’t changed.*

There were several comments criticizing an organizational leadership culture that did not support a psychosocial safety climate, psychological safety or well-being among the employees, and described experiences of not being heard:

*It took months before anyone even agreed to listen, - - that’s the impression that I got, that at the level of faculty management and higher they thought that the teaching personnel was just being difficult, they were being obstructive and were unwilling to learn a new system, or that this was resistance to change, or something like that. So it is very difficult, if the only way to be heard in a meeting like this is to have people on sick leave, and you need to talk like holding back your tears there, that is the way to be heard.*

*That kind of dishonesty of that jargon where you try to embellish certain things and you say that these are not problems these are challenges, these are positive opportunities for growth, - - it is really damned disrespectful of the university management to use that kind of language when they are trying to carry out a reform or more broadly in leading that organization - -. It alienates and feels arrogant and like primitive use of power where you systematically underestimate that community consisting of experts, whether they are researchers or administrative personnel they are all very highly trained experts, and then they are led in this kind of supercilious manner. - - it is completely useless to come and talk to people about their anxiety or what kinds of feelings they are having if you don’t come across as sincere. If you don’t seem like you actually care what the situation is, if you don’t care people are not going to tell you anything, they don’t put themselves on the line if you obviously haven’t put yourself on the line.*

One participant took the view that the focus of the leadership at the university should be generally better thought out for the specific demands of the work, and more human-centered, trustworthy and well-being supportive leadership would be needed for many reasons:

*- - in the university, where, after all, the output is people’s knowledge capital, creativity, courage, enthusiasm and motivation, so the goal of the leadership should be that it doesn’t die, that it stays and there’s honor and people dare to take more risks - - How it’s done, leading people and engaging them and cultivating those prerequisites; in my opinion there’s really a lot to do, we should somehow get rid of this kind of managerial, finances-driven, indicator-driven conversation - - we should take care of that motivation and enthusiasm staying there. Now that there’s so much of that malaise in our university as well, I think it’s precisely because we have failed in that leadership. All that kind of ceremonial talk that remains at the level of talk and if the actions don’t match with it, it is very unmotivating. And building an atmosphere of trust, or destroying it, which happens very fast, it is very, very important.*

Organizational practices in leading psychosocial safety climate and well-being also appeared to have an important role in enabling and supporting or challenging the supervisors’ own work in leading the same phenomena within their teams, but this support was not always forthcoming:

*- - if the mainstream culture were in accordance as well, - - it would be that organization’s way of functioning for real and not just in some ceremonial talk, in some slide set. That would of course be the biggest thing one could get. Otherwise you have to, there are some micro environments where there are different rules that apply than around it and it is always arduous and with what means do you set yourself against the mainstream culture - - that kind of clear support, that I notice what you’re doing and it’s really good that you do it, do keep doing it and tell me if you need anything—kind of signal is quite significant.*

*So as I said, I feel that at the immediate (leadership) level things are working very well, it’s just that there’s no support coming from the university management, on the contrary, they throw a spanner in the works.*

*Well, my most important function, as this term has already been used about these conversations, is to be a shit umbrella. The greatest threat to well-being comes from above, there’s all sorts of things leaking from there, and often they come like this needs to be ready in three days, and I try to filter them as best I can, so that that kind of endless interruption, endless bureaucratic waste of time would only be off my plate. So in my opinion a supervisor’s most central role is to enable success for their subordinates, and in our organization it means getting resources and protecting from things from above, that is sort of the absolutely most important role.*

Many supervisors and leaders found that they had had to cope with the challenges of remote working and remote leadership somewhat alone. It was a common experience that the organizational practices lacked deliberation, explicitness, and consistency: only few guidelines have been issued as to how to arrange work during the pandemic, and the supervisors themselves typically had limited time and opportunities to familiarize themselves with the topic and create specific courses of action.

In addition, some participants pointed out that the work included many aspects of uncertainty, and some were in the middle of an acute crisis, for example co-determination negotiations, and not knowing what kind of work they would be doing next and with whom. They also pointed out that uncertainty is a common underlying characteristic of university work, and, for example, fixed contracts undermine employees’ sense of security at a fundamental level.

### 3.3. Support for Working as a Supervisor

Overall, opinions about the sufficiency of supervisory support varied quite a lot—some were very happy with the diverse forms of support available to them while others felt rather alone in their supervisory work. Examples of support for working as a supervisor and for leading well-being were support from one’s own supervisor, systematic and clearly led forms of supervisory training and support, peer support, and instant support in acute challenging situations, as we describe below in more detail.

Albeit with some exceptions, many interviewees felt that they received little support from their own supervisor, and in some cases the connection was nearly nonexistent. For many supervisors the enforced remote work period had been very consuming, and some described being or having been pushed close to their limits. They were hoping for more support from their own supervisor and that there would be someone who would care for their well-being, too:

*At our workplace too, somehow my own supervisor’s support, there hasn’t really been any. - - I would have needed it at those times a bit, that they would have asked sometimes. You know, that someone had ever asked me how I was holding up.*

*Well of course that kind of support from above, that someone would support me in this supervisory work, it is virtually nonexistent [with a laugh]. Unfortunately. This middle management is quite an unfortunate level in that sense.*

Many participants also pointed out that the organization of supervisory support should be more systematic and clearly led. A common experience was that many forms of support are offered, but the information is fragmentary, and it is an ambitious idea to leave it to the supervisors themselves to first of all get a general picture of what is available and then make decisions on whether to participate. In most cases, workload and lack of time ended up preventing participation. Many noted that it would be very useful if it were more explicitly recommended and even compulsory for supervisors and leaders to attend certain forms of supervisory support and this was led in a centralized manner, in order to acquire the abilities needed and receive support for the supervisory work.

*I would say that there is not so much follow-up or encouragement that hey, do remember these things in your teams, that it is more like we are left quite alone, I don’t know if I just haven’t read some memos that I should have read, or I don’t know. - - So it’s assumed that we probably will notice this issue from all of that mass of messages, this part of well-being, like that.*

*If the supervisors’ training is left up to their own initiative, then I would argue that quite many of the supervisors don’t actively find their ways to any info meetings or trainings. There should probably be more precise announcements, that there’s this kind of event or training happening, that you are asked to participate.*

*I mean yes at least at the strategic level and at the level of training offerings and if I think about the mail for supervisors that we receive, things are very well and if I think about corona and leadership I was very pleased with it - - But maybe I think that the challenge is how people receive it. Since it comes by email or yeah, you can find it on the intranet. Hey, check out these training offerings, and when your work is like, khh khh khh (making a busy sound), schedule all the time and you run, you never get to finish that week’s work. So in my opinion the challenge is for people to get it that it’s necessary to invest in this, when you’re like, I, I can’t. - - for example in some [information removed for purposes of anonymization] system, you’re simply ordered, simply ordered, there they view it so that it is such an important issue, that now you know you’re going to this one. Sort of softly ordered, but in a way they clear a space for it there - - maybe over here there’s no such understanding that when you get into a supervisory position, you kind of need a driving license for it.*

*At some point I thought that it would be nice to go and have some kind of counseling (for the supervisory work) - - I don’t have time for such a thing. - - Lack of time is predominantly the reason why. Too much work.*

When asked about preferred forms of support for working as a supervisor, many participants pointed out that in their experience some form of peer support would be highly beneficial. Some were hoping that there would be some form of instant support, e.g., a leadership helpdesk, that one could contact consult especially with topical or acute challenging situations in supervisory work, either in the form of leadership counseling or a peer support group. Although supervisory support -related training and meetings were organized fairly frequently in larger groups, many felt that they would also have benefitted from encounters in smaller groups with opportunities for more concrete problem solving and exchange of good practices with other supervisors working in similar substance areas.

### 3.4. Characteristics Specific to Working in Academia

A pervasive aspect in the discussions about well-being at work was the specific characteristics of working in academia that affect both well-being and leading well-being at universities. Many participants pointed out that for many employees, the work is more than just work and thus their role and action something beyond that of a typical employee. In addition, the work includes specific aspects that make it particularly exacting, such as high standards, a demanding working culture, multiple roles, unpredictable schedules and changes, constant competition and the pressure to succeed. All this poses significant challenges to well-being. It appeared that the specific characteristics of academic work are a factor that affects in the background and challenges good practices of supporting well-being at the team level and at the organizational level, from both the employee and supervisor point of view.

*But in my opinion, no research is a nine-till five kind of a job. I think that we’re more like some kind of damned top athletes, you know, that we should be the first ones to publish in the world. And then we have the Academy (of Finland) funding battles that we should succeed in. And if I as a group leader prepare an application for the Academy, the number of working hours is pretty substantial. And where you take it from, for most, you take it from your free time, because the day is already quite burdened. - - And then if I think about, say, Iivo Niskanen (Finnish athlete), some skier, they’re not either like, oh, it’s 15.45, I will pack my skis now, I’m not training anymore. So in a way, you kind of have to do it, and there will be those pressure peaks too. There will be manuscripts and you’ll have a couple days to comment or something. And they are the kind that you’re never able to schedule them. They simply come when they come. - - So in that sense this is a very peculiar place to work, and this certain aspect, I don’t know if our construction workers, if they had to apply for money first so that they get to start building a house, it might not work.*

*It is though, in the academia, always that sort of balancing act, how close you are to burnout, are you a bit closer or a bit further away.*

In addition, in academia, the paths to supervisory roles are diverse, and this naturally also affects leadership practices. Some are very interested in the supervisory role and experienced in it, while for others it may come as a sudden additional duty while the main focus is on other things. Some had undergone extensive training while others had participated in none. Interestingly, many participants mentioned that they viewed their role as a supervisor mostly as a colleague, an enabler, and a coach—characteristics that partly emerge from the specific context and are also aligned with leadership approaches such as servant leadership or coaching leadership. One recurring feature among supervisors was that they had a very multifaceted role and were responsible for a long list of diverse tasks.

*The recruiting system in academia where you advance according to your academic merits into a position and after this it is assumed that these people would be capable supervisors, in reality they have been successful researchers, and a researcher’s job is clearly quite different from working as a supervisor, and in this case one might have, for example, utterly wretched social skills, and no background for it, leadership-related or even understanding of why it would be necessary.*

*- - traditionally it has been that someone is obliged to become a supervisor, but now there’s investment in it and someone might even want to take that position, so that is changing.*

*And then if you think about all the things that you are responsible for as an immediate supervisor. I am responsible for all the finances of my research projects, human resources, health and safety at work, exposure issues. - - And then they always say, this will only take, a new piece of work, it’ll take ten minutes. And if I ever get to talk to the university management, I’ll take the picture of Gulliver from my children’s book when he’s down and those Lilliputians’ ropes are there (holding him down). So it is just the same for myself, nothing takes a long time, but when you have enough of those things. Then you don’t have time to do them properly.*

From an organizational point of view, universities are rather fragmented in that they consist of many dissimilar units with differing characteristics and needs. Many participants pointed out that it was hard to lead such an organization or find ways that would work well for all concerned. There also seemed to be high appreciation for autonomy of the units to do what worked best for them. This definitely has its advantages, but the flip side may be the haphazard nature of leadership practices—if unified policies are lacking, the reality may vary from outstanding to poor and everything in between.

*- - that there’d be a shared experience for everyone that works here that you are genuinely cared for, I think that it is probably more challenging than in some enterprise. Simply due to the fact that at the university, people are committed to different kinds of things, you are committed to a research group or your field or the students or something, but few people are working there because of that institution. So it is different than in an enterprise - -. And it’s because of the structure of the organization. It’s because of those reasons that people are there for, and also because of how the whole thing is built. It is more challenging.*

*This is like, I wouldn’t want to lead this joint. It is so multi-dimensional, but there should be flexibility on the one hand for some wanting to go with the same rules for everyone. But it should be reflected on somewhere, at what point does the specificity of the field become an obstacle to it.*

### 3.5. Interconnections between the Main Categories

**Figure 1:**
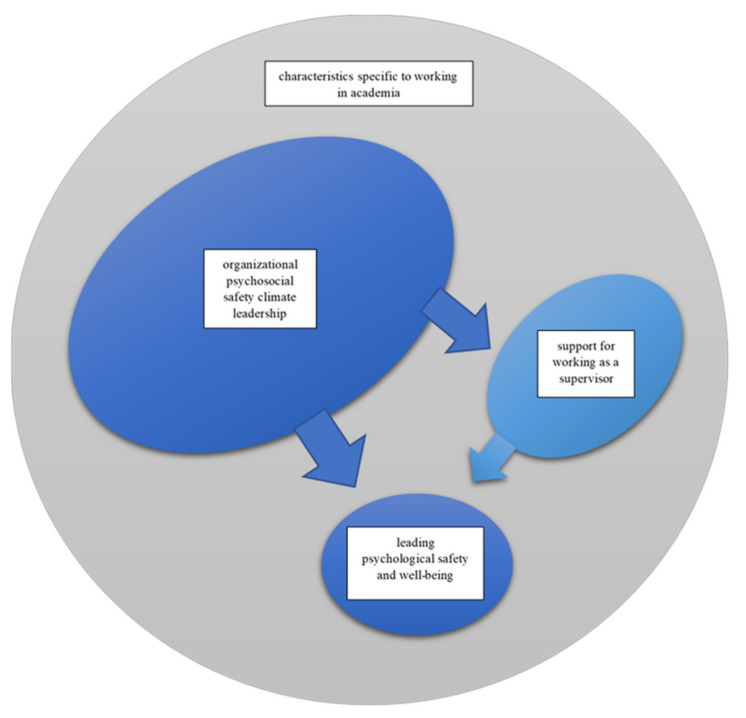
The interconnections between the main categories.

According to this analysis, the practices of leading psychosocial safety climate within the organization as well as practices of support for supervisors appeared to strongly influence supervisors’ practices of leading psychological safety within smaller units. The practicalities of supervisor support, such as what kinds of support was offered and how it was organized within the organization, seemed to be focal with regard to the skills and readiness of supervisors and leaders to act in ways conducive to a psychologically safe environment. Similarly, many aspects of psychosocial safety climate leadership within the organization, such as whether the supervisors were given enough time and resources to do the needed work, whether the supervisors’ well-being was supported, or whether the organizational practices were detrimental to employee well-being, appeared to have a significant role in the supervisors’ opportunities to do their own part well. In addition, the results suggested that the specific characteristics of working in academia were an underlying factor that influenced and at times challenged leading psychological safety and well-being in smaller units as well as leading psychosocial safety climate within the organization. These interconnections between the main categories and the phenomena studied are presented in Figure 1.

## 4. Discussion

This study focused on the central aspects of leading psychological safety and well-being in remote work conditions, both at the team level and the organizational level. The focus was on the experiences and perceptions of academic leaders and supervisors themselves, which has been a less common approach in the remote work literature and also overall in the present leadership research: most studies have focused on how employees perceive their leaders. This study examined the topic using qualitative methodology, which has been a less typical approach within PSC and psychological safety-related research. Moreover, remote work conditions have received little attention in the research on psychological safety and psychosocial safety climate, although these conditions were already common before the enforced remote work period and are likely to pose specific needs and challenges with regard to psychological safety [25,60]. Furthermore, psychological safety and psychosocial safety climate have very rarely been studied together within the same empirical study [40; for an exception, see 39], and therefore, this study also contributes significantly to understanding the interplay and reciprocal relations between these two focal working life phenomena. In the following, we will discuss these main findings and their theoretical and practical implications.

### 4.1. Main Findings

The analysis resulted in four main categories: (1) supportive and challenging aspects of leading psychological safety and well-being, (2) supportive and challenging aspects of organizational psychosocial safety climate leadership, (3) support for working as a supervisor, and (4) characteristics specific to working in academia (see Table 1).

Regarding central aspects of *leading psychological safety* remotely, the role of remote interaction was underlined in the participants’ views on the topic, both as a challenging aspect and as a source of supportive actions. It was frequently stated that maintaining a sufficient connection and supporting psychological safety remotely requires more time and effort than it does face to face, as well as intentionality as to what is pursued in each form of communication. Encounters and communication, either formal or informal, do not just happen fluently as a natural, embedded part of the working day as when working face to face, instead, they need to be planned and arranged in a very different way. Many participants pointed out that there is also much more uncertainty involved: it is hard to know how the employees really are at the other end of the line, and misapprehensions are generally more common when communicating remotely. The findings on remote leadership during the COVID-19 pandemic are similar: managers found that remote leadership was more time consuming and required more planning, communication was compromised, and it was more difficult to know how employees were and to maintain contact with them [31]. In our data, many had solved these challenges by investing more in personal communication with each employee, either by phone calls or video meetings. On the other hand, many supervisors felt inadequate regarding the time they could allocate for this purpose or consumed by how much time they had to invest in it.

Trust was also a prominent element of participants’ descriptions of the main aspects of leading psychological safety remotely. Many pointed out that trust needs to be a starting point in remote work in general: the work in these conditions is decidedly independent and dispersed, and supervisors need to trust the employees and their readiness to do their work in the best possible way, as opposed to trust being something that needs to be earned. Such positive expectations, trust, and respect are the bases for psychological safety [12,15]. Creating and showing trust as well as giving authority to employees has also been noted as a significant aspect of remote leadership in earlier research [44]. On the other hand, many participants pointed out that building trust and connection, especially with new team members, was more challenging remotely, because the slight nuances of interaction are missing and encounters with informal interaction which are generally very important for building rapport and trust tend to be much less than when working face to face. Many participants also noted that informal communication and emphasis on the human aspects of work are typically reduced in remote work and the role of interaction tends to be more functional and effective, and workdays overall more intense, which is also reported in earlier research [31].

The analysis suggests that many leaders perceived severe deficiencies in *leading psychosocial safety climate within the organization*. A common experience was that there was a marked discrepancy in PSC-related policies: employees were offered many means of support for well-being, but at the same time, major underlying issues that challenge well-being and the basic prerequisites for work, such as excessive workload and lack of resources, were ignored. Many stated that attention had not been paid within the organization to how extremely stressful the employees’ conditions were during the enforced remote work period, and the measures needed to ease the situation had not been taken; on the contrary, in some cases, the organization had added stressful aspects to the work. It appeared that the employees had had to deal with the profound crisis and threats of the pandemic, the unexpectedly altered remote working conditions (e.g., inadequate workstation and surroundings at home), added workload (e.g., shifting quickly into online teaching and making the necessary arrangements), and additional reforms taking place within the organization (e.g., new administrative practices), and in many cases, the workload and lack of resources was already a challenge prior to the specific challenges occasioned by the pandemic. The overall situation and the level of strain were deemed unreasonable, deplorable, and severely detrimental to the employees’ well-being, and in some cases actually making the employees ill. In many participants’ experiences, the organization had failed to take the needed actions and appeared untrustworthy and insincere. When comparing these results with the definition of PSC and its content domains [11], the participants indicated that there was senior management support for stress prevention, but not real management priority to psychological health and safety versus productivity goals or listening to the employees’ perspectives on PSC-related issues.

Furthermore, according to many participants’ descriptions, it seemed that the cohesion was lacking in leading psychosocial safety climate within the organization, and that the organization’s general line in leading well-being related issues remained obscure to the employees and even to the supervisors themselves. This may be related to the specific characteristics of university organizations, as they tend to be very large and rather fragmented than uniform [61], and remote work conditions may further exacerbate this challenge. In research focusing on the characteristics of university organizations, universities have been referred to with classic concepts such as organized anarchy [62] and loosely coupled systems [63], which refers to a lack of internal coordination and regulation. Even in more recent research, it has been stated that universities remain undermanaged institutions that still have diffuse structures of authority [64], and it is common for European universities to mix managerial and collegial forms of governance [65], which demonstrates the challenges of leading universities as a whole.

Finally, it is important to note that there was quite a lot of variation between the experiences of PSC among participants from different universities: even though in many cases the policies were experienced as insufficient or unacceptable, in some others the organizational leadership of PSC and well-being was perceived to be very good and comprehensive, and the organization seen as really striving to have it as a priority and acting in many meritorious ways. This is aligned with PSC being an organization-specific phenomenon that describes the climate within a given organization for employee psychological safety and health.

Overall, when discussing leading psychosocial safety climate at the organizational level, challenging aspects were much more underlined than supportive ones, whereas when discussing leading psychological safety at the team level, the participants offered rich descriptions of practices that supported it. This is not to say that leading it at the team level would not be demanding—indeed, one of the most prevalent comments on the topic was that leading psychological safety remotely was very challenging. Nevertheless, the participants had made great efforts and come up with many psychological safety- and well-being-supportive leadership practices during the enforced remote work period.

Many participants were hoping for better and more consistent *support for working as a supervisor.* In the participants’ reports, their work and the period of the pandemic especially, appeared to be very demanding, and many of them had had to manage the well-being related aspects of their supervisory work rather alone. Many were hoping for more peer support, which has also been related to effective and well-being supportive leadership in earlier research [66]. Overall, in many participants’ experience the organization seemed to lack cohesion in leading well-being related issues within the organization, and this made it more challenging for supervisors to do their own part well. Feeling supported in one’s supervisory position was arbitrary and, in many cases, thin. There are similar findings from other countries: academics feel unprepared for leadership in academia, and better leadership training opportunities and support are needed [67]. In addition, surprisingly many supervisors also felt that they had to actually protect the employees from additional burden coming from higher levels in the organization.

*Characteristics specific to working in academia* appeared to crucially influence leading both PSC and psychological safety. The working culture and conditions appeared wearing and mentions of excessive burden and burnout were frequent in the participants’ comments. More than one participant used the same description of academic work not being normal work, but more like academic athletics and top-level sports. Interestingly, earlier research has also noted that university employers seek “academic superheroes” who should excel in various different aspects of work [68]. As mentioned earlier, academic work also entails certain aspects that cause unpredictable workloads and stress. Academics face expectations from a range of actors, and changes in university funding arrangements and the competitiveness of academic labor markets, for example, have led to an increase in part-time, project-based, and fixed-term contracts in European universities and beyond [61,69]. Overall, academic jobs are increasingly insecure, more accountable, more entrepreneurial, and less well paid [23,70], and in Finland more specifically, academics are simultaneously highly satisfied and highly stressed [23]. In our data on supervisors and leaders in various and, in many cases, senior positions, the aspect of demanding, high-performance work combined with meager resources was prominent, and such working conditions are obviously stressful. In addition, in some ways there appeared to be two different realities and two different stories with different characteristics within the organizations: teaching and research personnel faced different core challenges than support services and faculty personnel, which can be challenging when leading the organization as a whole.

Overall, it appeared that even though familiarity with the topics studied differed between participants, even those participants who found it difficult to describe their practices related to them explicitly, seemed to have acted intuitively in many supportive ways. In Finland, power distances are generally low, and the culture entails aspects such as valuing interpersonal trust [71]. Finnish leadership has been described as consensual and informal and does not favor heroic leadership but rather viewing the organization as a system [72]. These underlying cultural aspects may also influence how interpersonal issues such as psychological safety are acknowledged, understood, taken into consideration, and valued within organizations and teams.

### 4.2. Implications

#### 4.2.1. Theoretical Implications

Earlier research has shown that psychological safety refers to a level of comfort or freedom from fear, whereas safety in PSC refers to freedom from psychological harm and injury [11]. This distinction was very noticeable in the data: in their descriptions of psychological safety, the participants described a number of practices that support good-quality connections between team members and the experience of the team being safe for interpersonal risk-taking, whereas the aspects mentioned in relation to PSC were such that largely affect psychosocial working conditions and in turn psychological health. Both aspects are fundamental to occupational well-being, and one central contribution of this study was to examine both phenomena in the same study.

Quantitative research has moreover framed PSC as an antecedent to team psychological safety [11]. Both PSC and team psychological safety may affect interpersonal factors, but PSC may additionally affect a range of other psychosocial hazard factors as well, such as work pressure and low job control, and general psychological health [11,13,40,50]. In our study it was apparent that PSC is not only related to the general psychosocial working conditions, but also appears to be a focal enabler, if not a necessity for psychological safety -supportive leadership to take place. This is a novel observation as there is very little research on PSC and immediate leadership [73], and in future, it would be interesting to study it further, also using quantitative methodology. We discovered that the practices of leading psychosocial safety climate within the organization as well as practices of support for supervisors appeared to directly influence supervisors’ practices and opportunities to successfully lead psychological safety within smaller units. Examples included having clear guidelines on how to act as a supervisor regarding psychological safety and well-being related issues, having enough time and resources allocated to accomplish this work, being able to work in a psychological safety-supportive way as a part of a deliberate and unified mode of action regarding this in the organization, having personal support from above similar to that being offered to the employees as a supervisor—or the opposite. At worst organizational practices impaired employee well-being and psychological safety, and under these circumstances it was very hard for the supervisor to remedy the overall situation.

In our data, both psychological safety and PSC appeared to include the focal element of trust, although this is not strongly emphasized in the literature theorizing PSC. Perhaps this is also a difference in the perspective and in the observer: from an organizational point of view, PSC is about psychological safety and well-being related commitment, priorities, policies, communication, participation, and involvement. From an employee point of view, the underlying quality of these actions also matters: if the actions, intentions and policies appear genuine and whole-hearted, and the organization appears trustworthy in truly having employee well-being as a priority. This is an interesting observation as earlier quantitative research represents the core characteristics of PSC and psychological safety as very distinct [39] and invites further research on the topic.

Finally, as employee well-being as a broad concept is an increasingly central challenge in today’s working life, it is nowadays often conceived of as a whole: leading well-being in organizations. It is worthwhile reflecting on whether PSC is or should be a distinct part of it, or if it often is intertwined with the broader well-being and the related policies in the organization. In the interview data, the participants’ perceptions of psychosocial safety climate leadership and of the leadership of more general aspects of occupational well-being appeared to often be closely intertwined.

#### 4.2.2. Practical Implications

In a remote setting, maintaining a good connection with the employees and supporting psychological safety requires specific effort and more time than when working face to face, as the opportunities for interaction do not occur naturally as an integral part of the working day but need to be deliberately created. Arranging sufficient time for leaders and supervisors to do this is a question of well-being both on the employee and the leader side—from employee and team perspective, sufficient support is imperative, and from a supervisor perspective, it cannot be achieved in additional working hours and at the expense of supervisor well-being.

This raises the question of prioritization and policy: which essential aspects of leadership today most urgently require the leader’s input and working hours? To set the priorities for the various kinds of supervisory duties within the organization, it is important to understand that in today’s working life, and in organizations such as universities in particular, the most valuable currency in most jobs is the human know-how, the shared learning and development of individual and team expertise [74,75]. For this to thrive, or even for any development and learning at all to take place, psychological safety is essential. As one interviewee aptly put it, under these circumstances the key focus regarding leadership within the organization should be on creating the prerequisites for human know-how, creativity, courage, motivation and engagement. In this nurturing the element of trust within the work environment is crucial.

Similarly, psychosocial safety climate is an extremely important aspect of organizational policy and practices in today’s working life, and leading it successfully requires clarity on priorities. It is essential that the importance of psychosocial health and psychologically sustainable work is fully understood and endorsed within the organization, and this is not always a given. It may be that there are contradictory views on priorities, and this may result in contradictory policies, which was apparent in many examples in our data. If this is the case, it likely will take time to establish an organizational culture that fully owns and endorses well-being as a first priority over productivity measures and understands the necessity of the former to the latter. Based on extensive research it is clear: the most common modern-day occupational injuries are mental overload, stress, and burnout [2,76], in particular because a growing proportion of employees are knowledge workers such as academics, having their mind as their primary tool for work. It is the employer’s duty as a part of their statutory labor protection policies to take good care of psychosocial health at work, but this aspect may still be less widely understood than more traditional aspects of occupational health, or there is considerable variation between workplaces with regard to this.

Our results demonstrate the importance of a good psychosocial safety climate within the organization as an imperative support for psychological safety in immediate work units. How well-being related issues are managed within the organization directly impacts the supervisors’ chances of doing their own work well. For example, if organizational policies such as inadequate resources, excessive workload or other additional strain are impairing employee well-being and safety, it is impossible for the supervisors to fix the situation within the team.

Leadership in the academic context has its characteristic features. As mentioned earlier, the pathways to supervisory positions are diverse, likewise the backgrounds for it [16,17,18]. According to our data, it would be important to ensure greater consistency in the training and support for supervisors, especially at the beginning of their supervisory duties, but also later on in the form of maintenance. Currently attendance in supervisory support or training is often left to the individual’s own discretion, which results in a wide variation in supervisory abilities and practices, from nonexistent to excellent or harmful and everything in between. With the often heavy workload and limited resources in everyday work routine, many supervisors also end up feeling that they need to prioritize something else even though they consider the topic important.

A prominent aspect of everyday work and well-being emerging in nearly all participants’ interviews was the challenge of insufficient resources and excessive workload per person. This is an issue that needs to be tackled better if universities truly want to give psychosocial safety and health top priority. Without firm policies regarding this, well-being related actions are really at risk of being mere ceremonial talk, as several participants described the situation. Academic work entails many unexpected elements that are hard to plan for in advance, but to some extent, so does any job in today’s working life. Work needs to be organized in such a way that the daily workload also leaves space and time for the unexpected, otherwise the work will be ineffective and unsustainable [77]. Overall, there seems to be an interesting discourse of normal rules not applying to the academic world, but this is not really the case—even in spite of specific challenges, many things can be done better, just like at any other workplace.

Overall, in this data, supervisory work appeared to be rather demanding, both in terms of its various challenging aspects, but also in terms of the amount of work. Additionally, more generally it has been noted that managerial reforms require academics to do an increasing amount of paperwork and teaching hours, and undertake more entrepreneurial activities and community service to meet the expectations of both their managers and external stakeholders, consequently academics’ workloads are increasing and many of them spend most of their weekends dealing with them [17,21,23,61,78,79,80]. Performance-based management and market-oriented managerial reforms are among the main sources of academic stress [23]. In our data, many supervisors had been and continued to be under a lot of strain, and some were also dealing with acute crises in the area of supervisory work without much support for solving the situation. One specific aspect of academic leadership is also the students: supervisors working in the teaching and research sector reported that the students are also an additional, invisible group of subordinates, since many of them also cared for their students’ well-being during the pandemic and in general, even though this did not show in their official job descriptions. Indeed, academic leadership typically involves multidimensional roles and tasks [16,17,18], and as one demonstration of this, many participants concluded that for them the working conditions during the pandemic had been much more peaceful and productive than usual since many of the nearly Kafkaesque aspects of working days had been diminished by simply not being at the workplace responding to various needs and requests. Despite the many challenges, many interviewees appeared highly committed to their work and emphasized this part of academic work in general: for many, its meaning is something more than just a job. Due to this, it is particularly important for the employer to set healthy boundaries for work and commit to supportive policies conducive to well-being within the organization.

In large organizations and especially in academic organizations, it is important to pay attention to well-being related issues as something truly shared and collective, and to have unified policies regarding them. According to the theory on psychosocial safety climate [11] and as shown in our data, mere organizational communication on these issues does not yet convey this if the various units remain fragmented in practice and sufficient organizational participation and involvement is lacking. Many participants pointed out that leading a university organization is challenging due to their multidimensionality. However, positive changes are possible, and many participants did also report ample changes in a positive direction regarding leading psychosocial safety and well-being within their organizations. The consistency and collectiveness in leading well-being-related issues within organizations are likely even more important in a remote work setting, where the employees are at greater risk of feeling isolated and alone with their work and issues.

### 4.3. Limitations

This study offers interesting novel information on leading PSC and psychological safety as well as their connection in an academic remote work context. However, the university context is in many ways different than in other organizations, for example, small private companies. Moreover, working in academia in Finland and its supervisor system entails aspects that may be partly different from arrangements in other countries [17,61]. Therefore, the results are partly specific to the context that they are derived from. Moreover, the study was carried out in enforced remote working conditions, and some aspects of the results are likely specific to these special conditions rather than generalizable to all remote or multi-locational work.

Participation in the study was based on voluntariness, and the interviewees were selected according to their willingness and interest to discuss the topics as well as their opportunity to attend. In interpreting the results, it should be born in mind that the individuals choosing to participate may have been particularly interested in the phenomena, or particularly frustrated by it. They may have been more familiar than others with well-being related issues and thus, poor leadership of psychological safety and well-being may be underrepresented in the data. This is of course a speculative issue, and one means to address the challenge of representativeness in this study was to form a sample with as diverse participants as possible in terms of their background in supervisory work and other differentiating aspects such as field or gender. In future it would be useful to combine different kinds of methodologies to gain further insight into the topic, and comparing the experiences in different subgroups could also bring additional value.

Finally, having multiple interviewers or analysts may add reliability by having multiple perspectives on the same data, but it may also impair reliability if the actions and principles are not aligned well enough so that all interviewees are treated equally in the process. Having one person conduct the interviews and the phases of the analysis can be beneficial in that it gives a detailed and comprehensive familiarity to the data. In this study, one researcher carried out the different phases of the qualitative research process in practice, but all parts of the study process and analysis were continuously discussed among all authors in order to improve credibility.

### 4.4. Summary, Conclusions, and Future Directions

The results of the study suggest that leading psychological safety remotely requires more time, effort, deliberation, and intentionality than when working face to face, and that the role of remote interaction is underlined in it. As to PSC, in many cases, the PSC-related actions and policies within the organization were felt to be contradictory and not genuine, and there were some severe deficiencies in leading psychosocial safety and well-being within the organizations. We discovered that academic work as a context entails many specific features that significantly affected the general picture and challenged the leadership of PSC and psychological safety in the organizations.

It is important to improve the cohesion in leading psychological safety and health within academic organizations; currently, university organizations may be somewhat fragmented and supervisors often quite alone in dealing with well-being related issues, and consequently, individual employees experience varying levels of support. As one part of increased cohesion in leading well-being in the university organizations, supervisory support should also be organized in a more consistent way.

Psychological safety and psychosocial safety climate are indispensable to today’s working life, where the most valuable currency in most jobs is the human know-how, shared learning, and the development of individual and team expertise, while for the employees their minds and mental capacity are their primary tools for work. In the context of academic work these aspects are particularly pertinent. How psychosocial safety is led in the organizations affects not only the general psychosocial working conditions and a range of psychosocial hazard factors, but also the preconditions for good leadership of psychological safety within smaller units in the organization. In order to fully own and endorse consistent PSC-supportive organizational leadership, the importance of the phenomenon needs to be well understood and acknowledged by senior management, which cannot yet be taken as a given in the diversity of working life. Employee health and well-being is not only an important value and priority in itself, but also an absolute necessity for employee productivity and success in today’s organizations.

## Figures and Tables

**Table 1:** Aspects relevant for leading psychological safety, psychological safety climate and well-being in enforced remote work in academia.

Main category	Generic Category	Subcategory
Supportive and challenging aspects of leading psychological safety and well-being	Supportive aspects	emphasizing trust
		investing in remote interaction
		being more deliberate
		cultivating authenticity
		nurturing an accepting environment
		providing sufficient task support
		ensuring the well-being of both employees and oneself
	Challenging aspects	limited interaction
		uncertainty of the employees’ situations
		increased intensity of work for employees
		own workload as a supervisor
		lack of time for maintaining sufficient contact
		varying employee competence in managing their own work and well-being
Supportive and challenging aspects of organizational psychosocial safety climate leadership	Supportive aspects	prioritization and comprehensive means of support from the organization for well-being related aspects of work
		regular opportunities for interaction with top management and healthcare services
		explicit guidelines and support for well-being related practices
	Challenging aspects	inconsistency in well-being related policies
		excessive workload and lack of resources
		straining organizational reforms
		unsupportive organizational leadership culture
		insufficient focus on essential aspects of leadership
		little support for or adding challenges to well-being-related supervisory work
		profound elements of uncertainty
Support for working as a supervisor		support from one’s own supervisor
		systematic and clearly led forms of training and support
		peer support
		instant support in acute challenging situations
Characteristics specific to working in academia		work role beyond that of a typical employee
		particularly exacting aspects of work
		diverse paths to supervisory roles and wide variation in the related skills
		fragmented organizations

## Data Availability

The data is available from Anne Mäkikangas upon reasonable request.

## Appendix A. Structure and Questions of the Semi-Structured Interviews

1. *Introduction to the topics and aims of the interview, going through ethical principles and defining the central concepts*
2. *Orientation and Context*

Please tell me a little bit about your current position and job description—what kinds of tasks and responsibilities do these entail?

What kinds of supervisory/leadership duties do you currently have? How many direct subordinates do you have and have there been changes in this during the pandemic?

3. *Own Well-Being as a Leader/Supervisor*

How have you personally experienced the long-standing enforced remote work?

How have you been during this time?

4. *Leading Well-Being and Psychological Safety*

How have you experienced leading remote work from the perspectives of occupational well-being and psychological safety?

In your supervisory and/or leadership duties, have you encountered challenges related to well-being and psychological safety among your subordinates?

If you have, how have you dealt with them?

With what kinds of concrete actions have you aimed to support the subordinates’ psychological safety?

As a supervisor/leader, what would help you in leading well-being and psychological safety? What kind of support would you hope for regarding this?

5. *Well-Being and Psychological Safety within the Organization*

If you think about the university as a workplace, how do you find that things related to well-being and psychological safety are taken care of?

How do these themes show in the everyday routines at the university?

Are employees and supervisors listened to regarding these issues?

6. *Overview, Conclusion, Assessment, and Future*

In what areas and issues related to remote leadership have you in your opinion succeeded?

Would you do something in your supervisory work differently if you now were to relive the time of remote work caused by the pandemic?

Do you have ideas or suggestions related to how the university could act even better in leading well-being and psychological safety?

We have come to the end of the interview—is there something that you would still like to add or share regarding your experiences as a supervisor/leader or regarding occupational well-being during the pandemic?
